# FTO-mediated m ^6^A demethylation of ULK1 mRNA promotes autophagy and activation of hepatic stellate cells in liver fibrosis: FTO regulates HSC activation by targeting ULK1

**DOI:** 10.3724/abbs.2024098

**Published:** 2024-08-22

**Authors:** Tingjuan Huang, Chunhong Zhang, Junjie Ren, Qizhi Shuai, Xiaonan Li, Xuewei Li, Jun Xie, Jun Xu

**Affiliations:** 1 Shanxi Key Laboratory of Birth Defect and Cell Regeneration Department of Biochemistry and Molecular Biology Shanxi Medical University Taiyuan 030001 China; 2 Department of Gastroenterology and Hepatology the First Hospital of Shanxi Medical University Taiyuan 030001 China; 3 Department of Cancer Radiotherapy Department Shanxi Provincial People’s Hospital Taiyuan 030001 China; 4 Department of Hepatopancreatobiliary Surgery the First Hospital of Shanxi Medical University Taiyuan 030001 China

**Keywords:** autophagy, hepatic stellate cells, FTO, m ^6^A methylation, ULK1

## Abstract

The activation of hepatic stellate cells (HSCs) is central to the occurrence and development of liver fibrosis. Our previous studies showed that autophagy promotes HSC activation and ultimately accelerates liver fibrosis. Unc-51-like autophagy activating kinase 1 (ULK1) is an autophagic initiator in mammals, and *N* ^6^-methyladenosine (m ^6^A) modification is closely related to autophagy. In this study, we find that the m ^6^A demethylase fat mass and obesity-associated protein (FTO), which is the m ^6^A methylase with the most significant difference in expression, is upregulated during HSC activation and bile duct ligation (BDL)-induced hepatic fibrosis. Importantly, we identify that FTO overexpression aggravates HSC activation and hepatic fibrosis via autophagy. Mechanistically, compared with other autophagy-related genes, *ULK1* is a target of FTO because FTO mainly mediates the m ^6^A demethylation of *ULK1* and upregulates its expression, thereby enhancing autophagy and the activation of HSCs. Notably, the m ^6^A reader YTH domain-containing protein 2 (YTHDC2) decreases *ULK1* mRNA level by recognizing the m ^6^A binding site and ultimately inhibiting autophagy and HSC activation. Taken together, our findings highlight m ^6^A-dependent *ULK1* as an essential regulator of HSC autophagy and reveal that ULK1 is a novel potential therapeutic target for hepatic fibrosis treatment.

## Introduction

Liver fibrosis is a complex physiological and pathophysiological condition following long-term chronic liver injury and is a necessary stage for various types of liver disease to develop into cirrhosis, liver failure and even liver cancer [1]. Cholestasis is an important factor in liver fibrosis, affecting approximately 200‒500 individuals per million inhabitants [2]. Currently, in addition to improving the etiology and reducing cholestasis, there are still some limitations in the treatment of hepatic fibrosis. Cholestatic liver disease is characterized by damage to the small intrahepatic biliary ducts, followed by proliferation and an inflammatory response, which leads to the activation of hepatic stellate cells (HSCs). Hence, the elimination of activated HSCs is recognized as the main strategy for preventing hepatic fibrosis.

Autophagy is a specific type of cell death that starts with the formation of double-membrane autophagosomes and ultimately fuses with lysosomal compartments to degrade cellular organelles and proteins [3]. Autophagy plays dual roles in hepatic fibrosis. A report showed that impairing autophagy could promote nonalcoholic liver fibrosis progression [4]. Additional studies demonstrated that autophagy in HSCs attenuated CCL4-induced liver fibrosis [5]. We previously reported that autophagy was upregulated in bile duct ligation (BDL)-induced hepatic fibrosis, causing the activation of HSCs, which ultimately accelerated liver fibrosis. Interestingly, a large number of autophagy-related genes (ATGs) are involved in autophagy; however, the specific ATGs regulating autophagy in cholestatic liver fibrosis remain unclear.

*N* ^6^-methyladenosine (m ^6^A) is a prominent dynamic mRNA modification that is modulated by methyltransferase complexes (writers), demethylases (erasers) and RNA-binding proteins (readers), influencing mRNA stability, translation, subcellular localization, and alternative splicing [6]. The “writer” proteins WTAP (Wilms’ tumor 1-associated protein), METTL3 (methyltransferase like 3), METTL4, and METTL14 catalyze m ^6^A methylation. In contrast, ALKBH5 (alkB homolog 5) and FTO (obesity-associated protein) function as “erasers” to reverse m ^6^A modifications. Additionally, m ^6^A reader proteins, such as YTHDC1/2 (YTH domain-containing protein 1/2) and YTHDF1/2/3 (YTH domain family 1/2/3), can bind to the m ^6^A motif to affect RNA stability or function [7]. Importantly, exploring the posttranscriptional regulatory effect of m ^6^A methylation on autophagy is helpful for identifying new diagnostic and therapeutic targets for cholestatic hepatic fibrosis.

Autophagy genes are closely associated with m ^6^A modification. A recent study reported that m ^6^A modification controlled autophagy by upregulating ULK1 (Unc-51-like autophagy activating kinase 1) protein abundance in HeLa cells [8], while another study showed that m ^6^A methylation controlled autophagy and adipogenesis by targeting ATG5 and ATG7 in 3T3-L1 preadipocytes [9], indicating that the ATGs targeted by m ^6^A methylation regulation may be distinct in different cells. However, it is unknown which ATG is targeted by m ^6^A methylation to induce autophagy, HSC activation and cholestatic hepatic fibrosis.

In the present study, we explored the potential mechanisms by which autophagy is regulated by m ^6^A during HSC activation. We found that the m ^6^A demethylase FTO promoted autophagy by recognizing the *ULK1* m ^6^A binding site, thus triggering HSC activation and eventually leading to liver fibrosis. Our study indicated that m ^6^A modification may be a novel and important posttranscriptional regulator of HSC autophagy and that ULK1 may be a potential therapeutic target for hepatic fibrosis treatment.

## Materials and Methods

### Animal experiments

Male C57BL/6 mice (20‒22 g) provided by the Animal Center of Shanxi Medical University (Taiyuan, China) were maintained and bred under normal conditions. The animal study was reviewed and approved by the Institutional Animal Care and Use Committee of Shanxi Medical University, and the license key was SCXK2021-0006. Two liver fibrosis models were established. Briefly, common bile duct ligation (BDL) was performed during laparotomy under isoflurane inhalation anesthesia by isolating and ligating the common bile duct ( *n*=8 per group). In sham animals, surgery was performed without ligation of the common bile duct ( *n*=8 per group). According to our previous report [10], cholestatic liver fibrosis developed approximately 4 weeks after the operation, and liver tissues were harvested and stored in formalin and liquid nitrogen. Another liver fibrosis model was generated by transducing FTO (0.1 mL of each at a titer of 2×10 ^9^ pfu) into liver tissue via the tail vein with adenovirus. The recombinant adenoviral vector encoding FTO (Ad-FTO) was synthesized by Sangon Biotech (Shanghai, China). This group of mice was sacrificed at 12 weeks ( *n*=8), and liver tissues were harvested.

### Cell culture and treatment

Immortalized mouse HSC JS-1 cells (provided by the Experimental Center of Science and Research of the First Hospital of Shanxi Medical University) were cultured in DMEM (Gibco, Grand Island, USA) supplemented with 10% FBS (Procell Life Technology, Wuhan, China), 100 U/mL penicillin, and 100 μg/mL streptomycin at 37°C in an incubator with 5% CO _2_. When approximately 80% of the JS-1 cells were adherent, AdFTO was transfected for 24 h, and then the cells were starved with serum-free DMEM for another 24 h. After FTO siRNA (Sangon Biotech) was transfected into JS-1 cells with LipoFiter ^TM^ 3.0 (Hanbio, Shanghai, China) for 6 h, the cells were cultured under starvation conditions for 48 h. To investigate whether FTO regulates autophagy and the activation of HSCs by targeting ULK1, we added Ad-FTO to the cells for 24 h after the start of ULK1 siRNA (Sangon Biotech) transfection for 6 h, followed by starvation treatment for 24 h. *In vitro* analysis of each group was performed in triplicate. The corresponding sequences are as follows: siFTO sense 5′-GAGCAGCCTACAACGTGACTTTGCT-3′, and antisense 5′-GAGTGCTCAACAGGCACCTTGGATT-3′; siULK1 sense 5′-CCUGGUCAUGGAGUAUUGUAATT-3′ and antisense 5′-UUACAAUACUCCAUGACCAGGTT-3′; and siNC 5′-GGTGAAGGTCGGAGTCAACG-3′.

### Cell viability assay

Cell viability was determined by CCK8 assay following manufactory’s manual. JS-1 cells were seeded in 96-well plates at a density of 4×10 ^3^ cells/well, and then treated with starvation for 6 h, 12 h, 24 h, 48 h. Subsequently, each well was added with 10 μL CCK8 reagent (Beyotime) and incubated 2 h. The optical densities of the wells were read on a microplate reader (SpectraMax M5; Molecular Devices, Sunnyvale, USA) at 450 nm. The percentage of viability was calculated by the ratio of experimental cells to normal cells.

### Antibodies

Primary antibodies against α-SMA (ab124964, 1:1000), collagen I (ab260043, 1:1000), LC3B (ab48394, 1:1000), and SQSTM1/P62 (ab91526, 1:1000) were obtained from Abcam (Boston, USA). Antibody against FTO (sc-271713, 1:500) was obtained from Santa Cruz Biotechnology (Santa Cruz, USA).

### Histological analysis

Liver tissue samples were paraffin embedded, and 4 μm tissue sections were stained via standard methods and subjected to histopathological analysis according to our previous reports [10]. Briefly, the sections were fixed with 4% paraformaldehyde, dewaxed with xylene, hydrated with alcohol, and boiled in sodium citrate buffer to retrieve antigens. Hepatic morphology and liver fibrosis were examined by H&E (Beyotime, Shanghai, China), Masson (MST-8003; MXB Biotechnology, Beijing, China) and immunohistochemical staining. For immunohistochemical staining, the sections were incubated with primary antibodies at 4°C overnight and then with HRP-conjugated secondary antibodies (Beyotime) at 37°C for 20 min. Finally, staining was detected using DAB (Beyotime).

### Western blot analysis

The BCA colorimetric method was used to quantify the protein concentration. SDS loading buffer (4×) (P1015; Solarbio, Beijing, China) was added to the samples, which were subsequently boiled for 10 min. Equal amounts of protein samples were separated via agarose gel electrophoresis and transferred to PVDF membranes (Bio-Rad, Hercules, USA). After being blocked with 5% dry milk for 2 h, the membranes were incubated with primary antibodies at 4°C overnight and then with the corresponding secondary antibodies (C05-07002; Bioss, Beijing, China) at room temperature for 1‒2 h. All the primary antibodies were diluted at a concentration of 1:1000. Proteins were detected using enhanced chemiluminescence (ECL) reagent (Bio-Rad).

### RNA extraction and quantitative real-time PCR

RNA was extracted using TRIzol reagent (15596018; Invitrogen, Carlsbad, USA) per the manufacturer’s instructions, and 1 μg of RNA was reverse transcribed using a cDNA reverse transcription kit (RR037A; Takara, Tokyo, Japan). The final cDNA samples were subsequently subjected to quantitative real-time PCR with a SYBR Green PCR kit (RR820A; Takara). The primers used were designed based on the gene sequences provided by Sangon Biotech. The target gene primer sequences are provided in Table 1.

**Table:** **Table 1** Sequences of the primers used in this study

Gene	Forward primer (5′→3′)	Reverse primer (5′→3′)
*FTO*	GCAGCTGAAATACCCTAAACTG	AGTCTGGTGTTCAAGTACTTGT
*ALKBH5*	CGGGACCACCAAGCGGAAATAC	CCTCTTCCTCCTTCTGCAACTGATG
*METTL3*	CGCTGCCTCCGATGTTGATCTG	TCTCCTGACTGACCTTCTTGCTCTG
*METTL4*	TGGGTGGTTACTGGATCATCT	AGCAAAGCACATAGCAGGAGC
*METTL14*	AGCAGACATAGAAGCCTTTGACATCAG	CCCAGGTCCAGCATTTCTCATTCG
*WTAP*	GCCTGGAAGTTTACGCCTGATAGC	TCTTGGTTCTCCTGGATAAGCATTCG
*LC3B*	TGTGTCCACTCCCATCTCCGAAG	CCATTGCTGTCCCGAATGTCTCC
*SQSTM1*/ *p62*	AGGAGGAGACGATGACTGGACAC	TTGGTCTGTAGGAGCCTGGTGAG
*α-SMA*	CTCCATCGTCCACCGCAAAT	GGCCAGGGCTACAAGTTAAGG
*Collagen I*	GCTCCTCTTAGGGGCCACT	ATTGGGGACCCTTAGGCCAT
*ULK1*	TTCAGCACCAGCCGCATTACG	CAAAGCCAGCAGAGGGAGCAATC
*Atg4b*	TGGAGTCAGAGAGGCACTGTAACG	TGTCTGTCAGTCCCAGGCGAAG
*Atg9a*	CAAGCCCGCCTCCAAGTACATG	TCCACAGCCAACACATCTTCATCG
*Atg12*	CGGACCATCCAAGGACTCATTGAC	TGGGGAAGGGGCAAAGGACTG
*Atg13*	ACCGATTGTCACTGCTGCTGAAG	TGCCCTTGCTTCCTGGAGAGTC
*Atg14*	AAGGAGAAGATTCAGCGGCACAAC	CATTGGGAAGATGACAGAGGTGAGC
*Atg16L1*	CAAGCCGAATCTGGACTGTGGATG	CGGTCGTGACTTCCTGAGACAATC
*YTHDF1*	CCCTGTCCTGGAGAAACTGAAAGC	GTACTTGATGGAGCGGTGGATGTC
*YTHDF2*	GGCAGTGGACACTTCTGTGG	TGTCGCAGTTGGCTATTGGG
*YTHDF3*	AATGCTTATGCTGGTGTCTGGTCTC	GTGTCCCTTGAATTGGTTACTGGTTTG
*YTHDC1*	AGGAAGAGGATGAAGAGGAAGAAGAGG	AAGGAAACAGACTCGGAACCAGAATC
*YTHDC2*	CCTGTTACTGTCCTGGTGTTCTGTG	CTTCCATCTCACTGTCACTGCTGTC
*GAPDH*	AGTCTGTGTAGTTAGAAGCTCCA	TGGTCCAGGGTTTCTTACTCC

### Immunofluorescence microscopy

The cells were fixed with 4% paraformaldehyde for 15‒20 min, permeabilized with 0.1% Triton X-100 for 15 min, and then blocked with 1% BSA at room temperature for 30 min. Next, the cells were incubated with primary antibodies at 4°C overnight, followed by incubation with FITC-labeled secondary antibodies (Beyotime) at room temperature for 1 h. DAPI (C0065; Solarbio) was used to stain the nuclei. An inverted fluorescence microscope (Olympus, Tokyo, Japan) was then used to observe the fluorescence.

### MeRIP-qPCR

A Magna MeRIP ^TM^ m ^6^A kit (17-10499; Merck, Darmstadt, Germany) was used for identification and transcriptome-wide profiling of m ^6^A. First, RNA is chemically fragmented into 100 nucleotides or smaller fragments, followed by magnetic immunoprecipitation with a monoclonal antibody against m ^6^A (ab284130; Abcam). After immunoprecipitation, the isolated RNA fragments were subjected to RT-qPCR.

### m ^6^A sequencing

m ^6^A-specific antibodies are most often necessary for high-throughput sequencing. m ^6^A sequencing was performed at Guangzhou Kidio Biotechnology. Total RNA was extracted with TRIzol, fragmented and immunoprecipitated with an m ^6^A antibody according to the instructions of the Magna MeRIP ^TM^ m ^6^A kit, after which the isolated RNA fragments were subjected to RNA sequencing.

### Quantification of m ^6^A RNA

To detect the m ^6^A RNA methylation status directly, total RNA was isolated and quantified using an EpiQuik m ^6^A RNA Methylation Quantitative kit (P-9005-48; Epigentek, Farmingdale, USA). Relative m ^6^A RNA methylation levels were then calculated using the formula provided by the manufacturer’s instructions.

### Transmission electron microscopy

The cells were fixed with 2% glutaraldehyde for 2 h. After wash with sodium arsenate for several hours, the cells were fixed with 1% osmic acid for more than 2 h. A gradient of acetone was used for dehydration at 5‒15 min per stage, and the mixture of acetone and embedding agent was soaked at room temperature for 2‒4 h. The epoxy resin was embedded for 24 h at 40°C and polymerized for 48 h at 60°C. Ultrathin slices were obtained with an ultramicrotome (LKB, Bromma, Sweden), placed in uranium acetate and lead citrate dye for 15‒30 min, and then examined with a transmission electron microscope (JEM-2100; JEOL, Tokyo, Japan).

### Statistical analysis

Data are expressed as the mean±standard error of the mean (SEM), and all the calculations were performed using GraphPad Prism 8.0 (GraphPad software, La Jolla, USA) or SPSS 23.0 statistical software (SPSS, Chicago, USA). In addition, statistical significance was evaluated using Student’s *t* test or one-way analysis of variance. *P*<0.05 was considered to indicate statistical significance.

## Results

### FTO is upregulated during HSC activation and BDL-induced hepatic fibrosis

**Figure 1:**
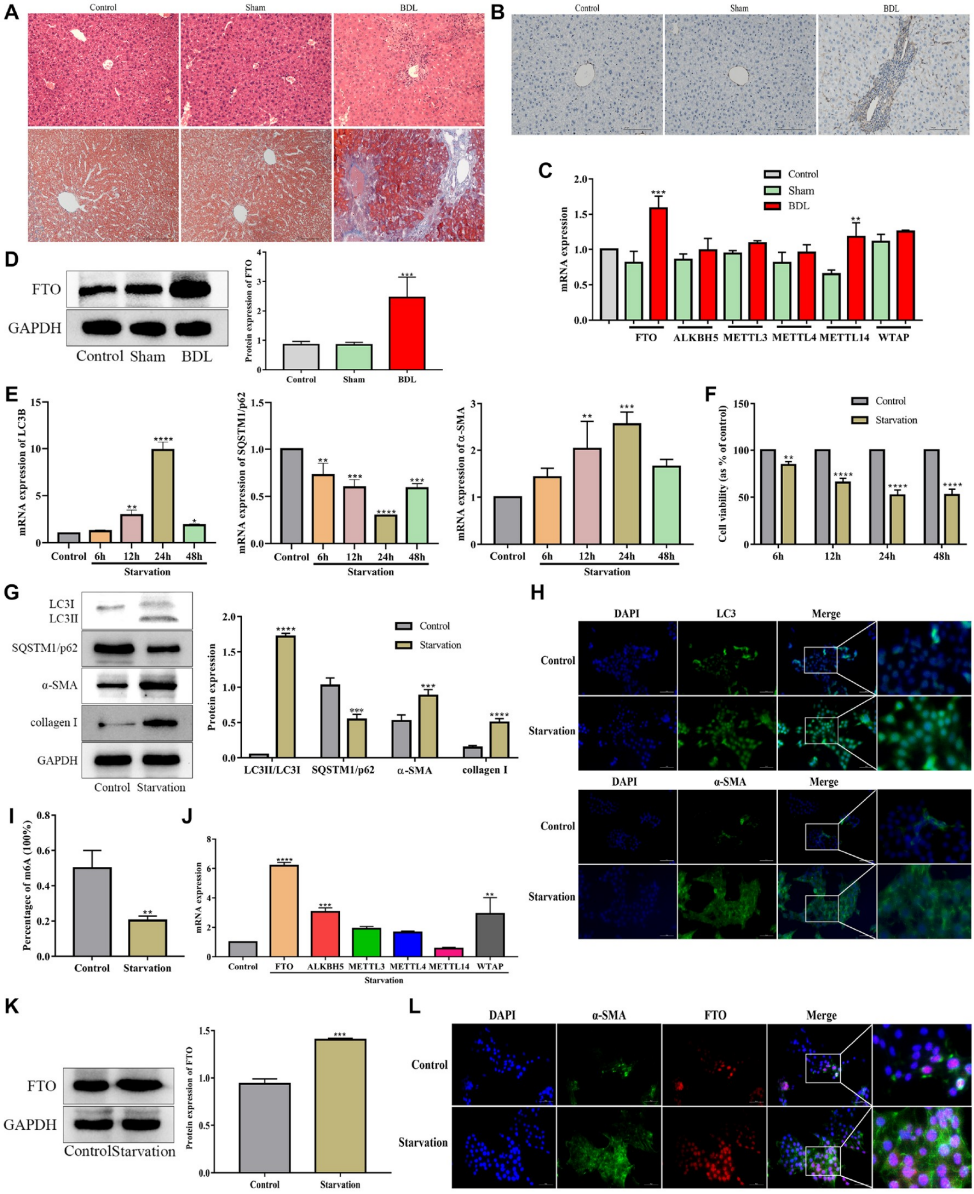
Figure 1 FTO is upregulated during HSC activation and BDL-induced hepatic fibrosis (A) HE and Masson staining were performed to evaluate the establishment of a BDL-induced hepatic fibrosis model. Scale bar: 100 μm. (B) α-SMA was semiquantitatively detected by immunohistochemical staining. Scale bar: 100 μm. ** P<0.01 vs Sham. (C) mRNA levels of m 6A writers and erasers were detected by RT-qPCR in mice with BDL-induced hepatic fibrosis. ** P<0.01 vs Sham. (D) Protein expression of FTO in the BDL-induced hepatic fibrosis model mice determined by western blot analysis. ** P<0.01 vs Sham. (E) RT-qPCR analysis of autophagy-related mRNA expression and HSC activation in JS-1 cells subjected to starvation at different time points. ** P<0.01 vs the control group. (F) Effect of starvation on the viability of JS-1 cells. ** P<0.01 vs Control. (G) Western blot analysis of autophagy and HSC activation in JS-1 cells subjected to starvation. ** P<0.01 vs the control group. (H) Immunofluorescence of LC3B and α-SMA in JS-1 cells subjected to starvation. The cells were stained for LC3B (green), α-SMA (green) and DAPI (blue). Scale bar: 20 μm. (I) The m 6A levels were detected by an EpiQuik m 6A RNA Methylation Quantitative kit. ** P<0.01 vs Control. (J) mRNA levels of m 6A writers and erasers were detected by RT-qPCR in vitro. ** P<0.01 vs Control. (K) Protein levels of FTO in JS-1 cells subjected to starvation were measured by western blot analysis. ** P<0.01 vs Control. (L) Co-localization of FTO and α-SMA determinded by immunofluorescence of FTO and α-SMA in JS-1 cells subjected to starvation. The cells were stained for FTO (red), α-SMA (green) and DAPI (blue). Scale bar: 20 μm. Data are presented as the mean±SEM. n=8 in vivo, n=3 in vitro.

Similar to our previous research methods, cholestatic liver fibrosis was induced by BDL [11]. HE staining of livers from BDL-treated mice revealed increased dilation of the bile duct, proliferation of small bile ducts, and infiltration of inflammatory cells in the portal area and around the bile duct, and Masson staining revealed collagen deposition in the BDL group ( Figure 1A). Moreover, immunohistochemical staining revealed that BDL increased the expression of α-SMA ( Figure 1B). These results suggested that the BDL-induced hepatic fibrosis model was successfully established. In addition, to explore the potential of m ^6^A modification in cholestatic liver fibrosis, we first used qPCR analysis to compare the mRNA expressions of m ^6^A writers and erasers. Interestingly, FTO expression was most obviously increased in BDL-induced hepatic fibrotic tissue, and FTO protein and mRNA levels were increased ( Figure 1C,D).

HSCs activation is the central link in the occurrence and development of liver fibrosis. To determine whether FTO is involved in HSC activation, we verified it *in vitro*. Autophagy is known to occur during starvation [ 12, 13] . Consistent with the literature [14], our results showed that autophagy was increased by starvation and peaked at 24 h in JS-1 cells ( Figure 1E), while cell viability was decreased ( Figure 1F); these findings were used for subsequent experiments. Western blot analysis revealed that the ratio of LC3 II/I was increased, while p62 was decreased in the starvation group at 24 h, further suggesting that autophagy was enhanced by starvation ( Figure 1G). Moreover, the expressions of α-SMA and collagen I were dramatically increased by starvation, indicating that starvation induced HSC activation ( Figure 1G). Immunofluorescence staining showed the same results ( Figure 1H). Subsequently, we examined the levels of m ^6^A modification during HSC activation, and m ^6^A RNA methylation quantification revealed a significant decrease in m ^6^A levels after starvation ( Figure 1I). As expected, consistent with the *in vivo* results, FTO exhibited the most obvious increase in starvation-induced HSC activation ( Figure 1J,K). Finally, we conducted immunofluorescence colocalization of FTO and α-SMA, and the results showed that the main regions with upregulated FTO were highly coincident with HSC regions ( Figure 1L). These results indicated that FTO may play an essential role in the process of HSC activation.

### FTO promotes HSC activation, hepatic fibrosis and autophagy

**Figure 2:**
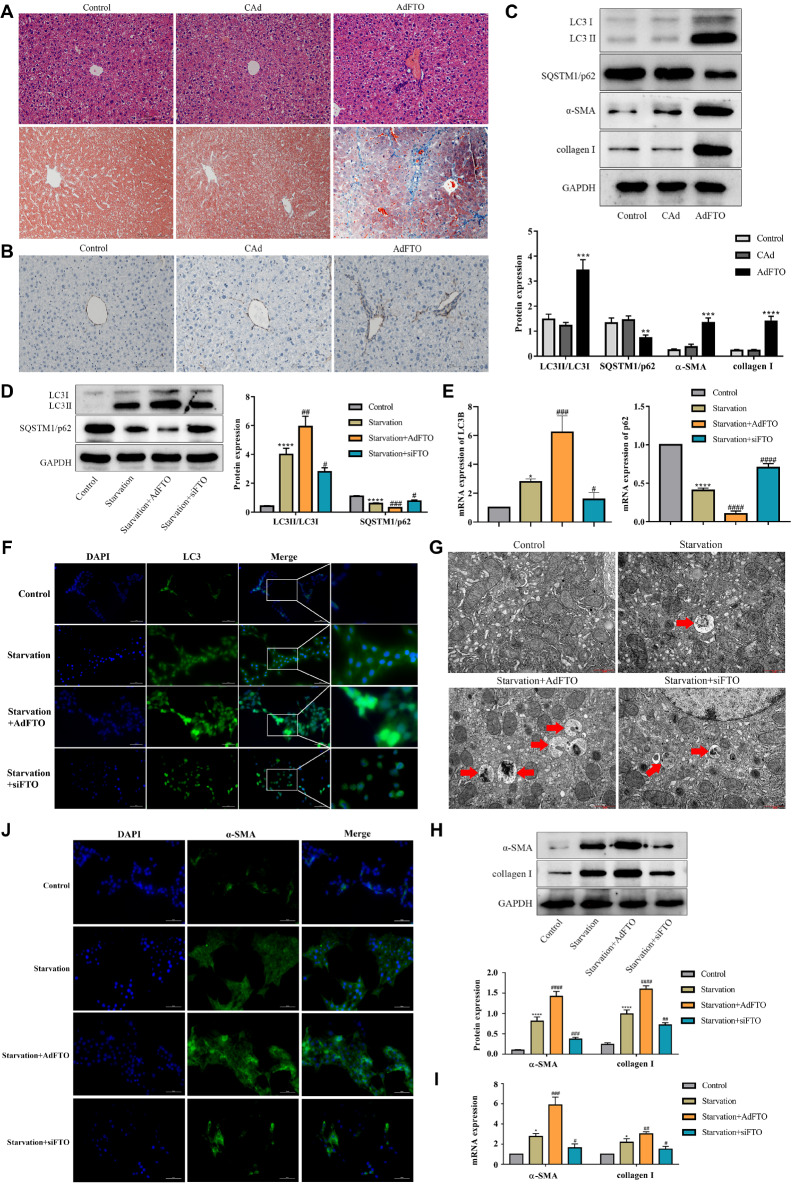
Figure 2 FTO promotes HSC activation and hepatic fibrosis as well as autophagy (A) The pathological changes in livers from mice treated with FTO were monitored by HE and Masson staining. Scale bar: 100 μm. (B) α-SMA expression in the liver by FTO was semiquantitatively detected by immunohistochemical staining. Scale bar: 100 μm. ** P<0.01 vs CAd. (C) Western blot analysis of the protein expressions of LC3B, p62, α-SMA, and collagen I in the livers of mice transfected with AdFTO. ** P<0.01 vs CAd. (D,E) Protein and mRNA levels of autophagy-related genes determined by western blot analysis and RT-qPCR in starvation-induced JS-1 cells transfected with AdFTO or siFTO. ** P<0.01 vs control, ## P<0.01 vs starvation. (F,J) Immunofluorescence of LC3B and α-SMA in starvation-induced JS-1 cells transfected with AdFTO or siFTO. The cells were stained for LC3B (green), α-SMA (green) and DAPI (blue). Scale bar: 20 μm. (G) Transmission electron microscopy was used to assess autophagosomes. Scale bar: 1 μm. (H,I) Protein and mRNA levels of α-SMA and collagen I determined by western blot analysis and RT-qPCR in starvation-induced JS-1 cells transfected with AdFTO or siFTO Data are presented as the mean±SEM, n=8 in vivo, n=3 in vitro. ** P<0.01 vs control, ## P<0.01 vs starvation.

To verify the role of FTO in hepatic fibrosis *in vivo*, we first transfected AdFTO into the mouse liver. HE and Masson staining revealed that FTO enhanced inflammatory cell infiltration and collagen deposition in the portal area ( Figure 2A). Western blot analysis revealed that the expressions of α-SMA and collagen I in the AdFTO group gradually increased ( Figure 2C), which was consistent with the immunohistochemical staining results ( Figure 2B). Our previous studies showed that enhanced autophagy accelerated hepatic fibrosis [10]. In this study, we hypothesized that autophagy is essential for FTO-induced hepatic fibrosis. To test this hypothesis, we detected the level of autophagy after FTO treatment. As expected, the conversion of LC3 I to LC3 II in the AdFTO group gradually increased, while that of p62 decreased ( Figure 2C), suggesting that autophagy was enhanced by FTO.

Is autophagy involved in FTO-induced HSC activation *in vitro*? To clarify this possibility, AdFTO and siFTO were transfected into starvation-treated JS-1 cells. As shown in Figure 2D, FTO markedly increased the starvation-induced ratio of LC3 II/LC3 I and decreased p62 expression, while siFTO reversed these changes, which was consistent with the RT-qPCR results ( Figure 2E). Moreover, immunofluorescence staining suggested that the level of LC3 was increased by AdFTO and decreased by siFTO ( Figure 2F). In addition, transmission electron microscopy confirmed that AdFTO increased the number of autophagic lysosomes, while siFTO decreased this number ( Figure 2G). Subsequently, HSC activation markers were detected. The results suggested that the ability of starvation to promote α-SMA and collagen I was further strengthened by AdFTO and weakened by siFTO ( Figure 2H,I). Immunofluorescence staining of α-SMA showed the same result ( Figure 2J). These findings suggested that FTO promotes HSC autophagy and activation.

### FTO promotes HSC activation via autophagy

**Figure 3:**
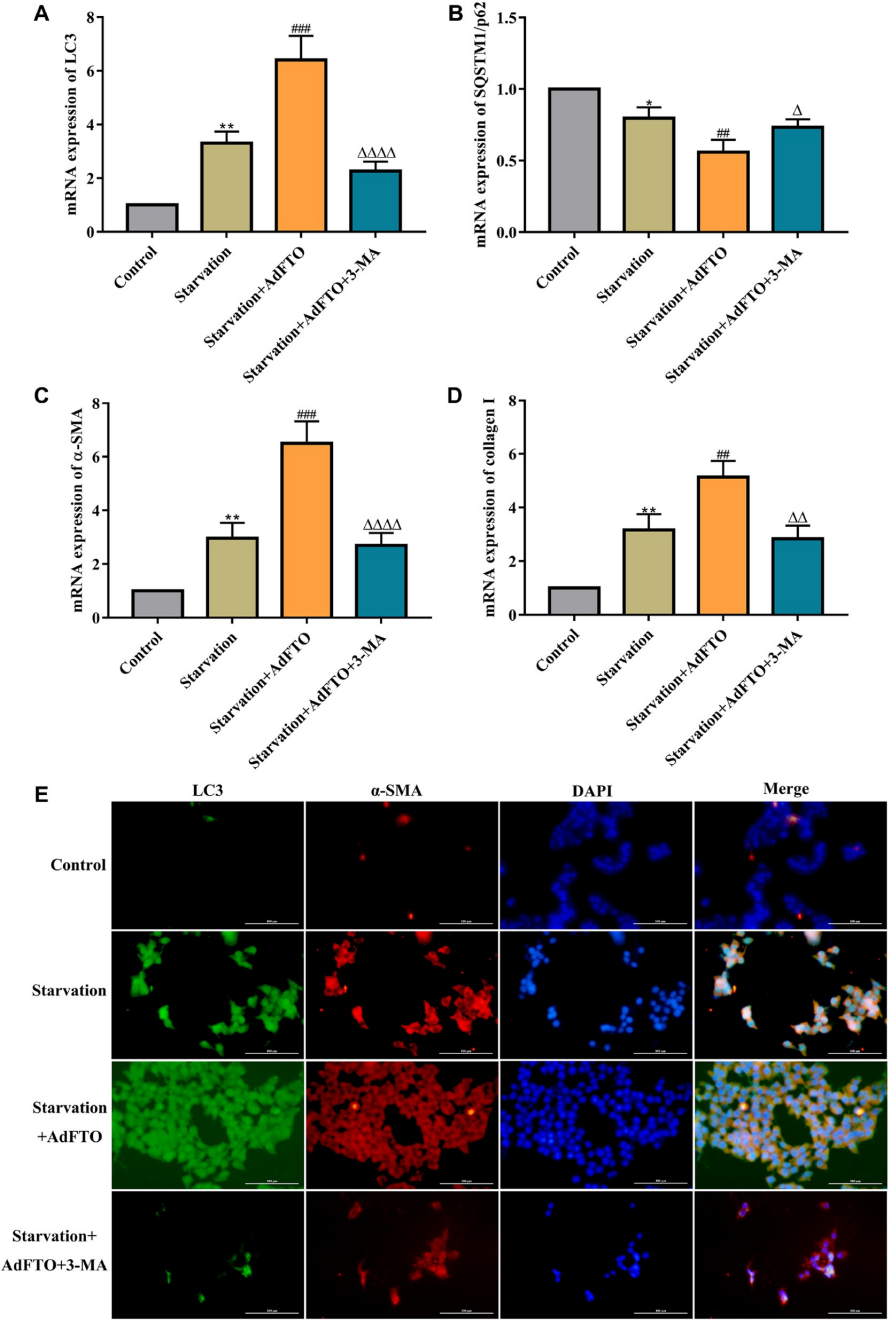
Figure 3 FTO promotes HSC activation via autophagy (A‒D) RT-qPCR analysis of the mRNA levels of LC3, SQSTM1/p62, α-SMA and collagen I in starvation-induced JS-1 cells after AdFTO treatment in the presence or absence of 3-MA. (E) Coimmunofluorescence staining of α-SMA and LC3 in starvation-induced JS-1 cells after AdFTO treatment in the presence or absence of 3-MA. Data are presented as the mean±SEM, n=3 in vitro. ** P<0.01 vs Control, ## P<0.01 vs Starvation, ΔΔ P<0.01 vs Starvation+AdFTO.

To verify whether autophagy is required for FTO-induced HSC activation, the autophagy inhibitor 3-MA was used in JS-1 cells in the presence of FTO. RT-qPCR results revealed that 3-MA significantly reduced FTO-induced LC3 upregulation and further increased FTO-induced p62 expression ( Figure 3A,B), indicating that 3-MA suppressed the autophagy-inducing effect of FTO. To further understand how autophagy impacts HSC activation, we examined the role of the autophagy inhibitor 3-MA in FTO-induced HSC activation. The results showed that 3-MA treatment markedly reduced α-SMA and collagen I mRNA expressions ( Figure 3C,D), suggesting that reducing autophagy affects the ability of FTO to promote HSC activation. Coimmunofluorescence staining of α-SMA and LC3 revealed the same results ( Figure 3E). Based on the above results, autophagy is required to mediate FTO-induced HSC activation.

### FTO mainly mediates the m ^6^A demethylation of *ULK1* to drive autophagy

**Figure 4:**
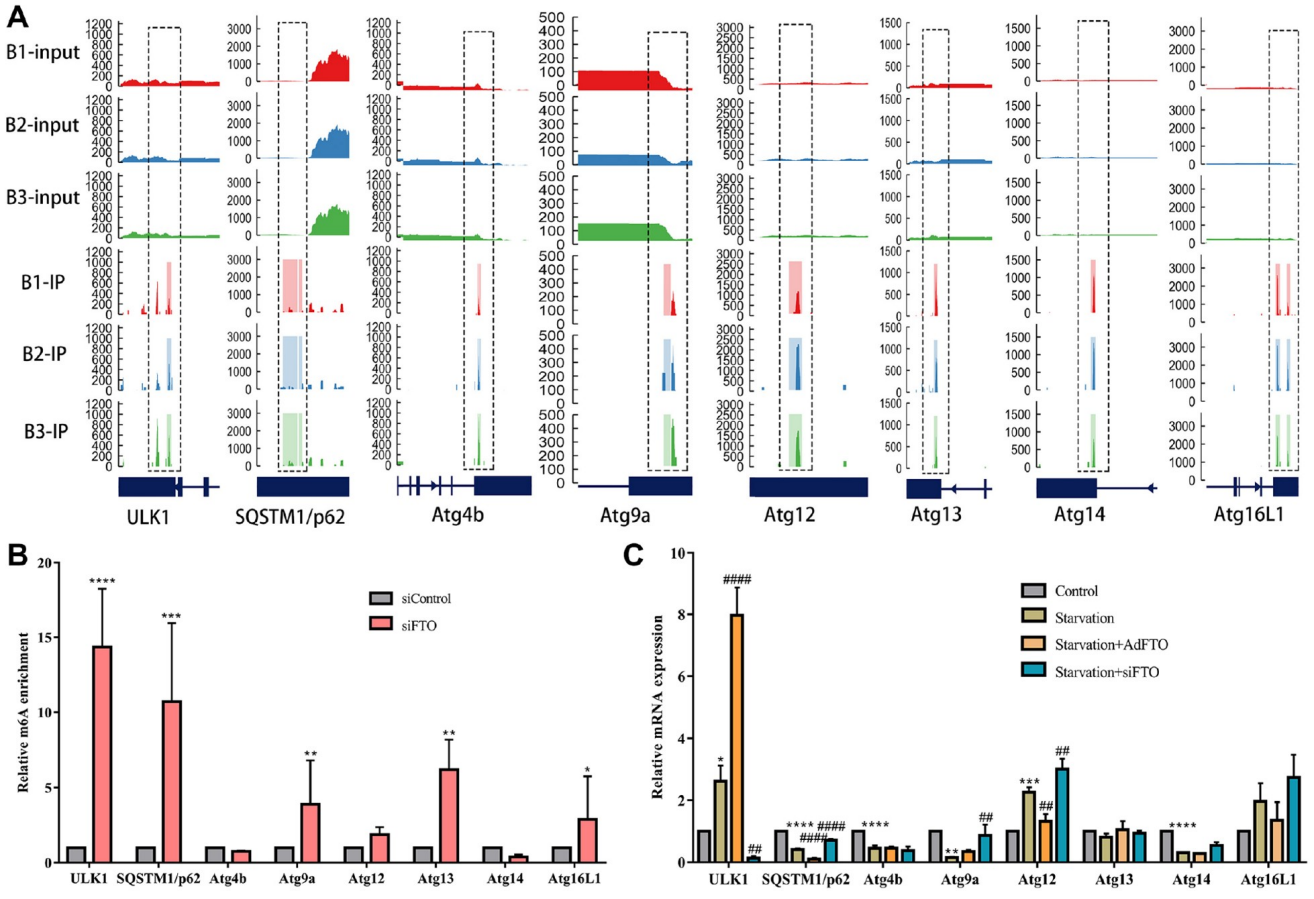
Figure 4 FTO mainly mediates the m6A demethylation of *ULK1* to induce autophagy during HSC activation (A) m 6A-seq of m 6A peaks at the ULK1, SQSTM1/ p62, ATG4b, ATG9a, ATG12, ATG13, ATG14, and ATG16L1 mRNAs. On the X-axis, the blue boxes represent exons, and the blue lines represent introns. The Y-axis shows the sequence read number. (B) MeRIP-qPCR analysis of the m 6A levels of the ULK1, SQSTM1/ p62, ATG4b, ATG9a, ATG12, ATG13, ATG14, and ATG16L1 mRNAs in the control and siFTO cells. ** P<0.01 vs siControl. (C) mRNA expression of ULK1, SQSTM1/ p62, ATG4b, ATG9a, ATG12, ATG13, ATG14, and ATG16L1 in starvation-induced JS-1 cells transfected with AdFTO and siFTO. ** P<0.01 vs control, ## P<0.01 vs starvation. Data are presented as the mean±SEM. n=3 in vitro.

We performed m ^6^A sequencing (m ^6^A-seq) of major ATGs to further explore which ATGs exhibit m ^6^A modification in BDL-induced hepatic fibrosis. A total of 8 ATGs, including *ULK1*, *p62*, *ATG4b*, *ATG9a*, *ATG12*, *ATG13*, *ATG14*, and *ATG16L1*, had m ^6^A modification sites ( Figure 4A). Then, to identify the target genes of FTO involved in autophagy, siFTO was transfected into JS-1 cells, and all ATGs with m ^6^A modification were detected by MeRIP-qPCR. The results indicated that knockdown of *FTO* significantly increased the m ^6^A level on the mRNA transcripts of *ULK1*, *p62*, *ATG9a*, *ATG13*, *ATG16L1*, and especially *ULK1* ( Figure 4B). More importantly, to determine which ATG was the direct target of FTO, AdFTO and siFTO were transfected into JS-1 cells followed by starvation. Interestingly, we found that compared with those in the starvation group, the protein levels of ULK1 in the FTO-overexpressing group were markedly increased, while those in the *FTO-*knockdown group were decreased ( Figure 4C). Other ATGs, such as *ATG9a*, *ATG13*, and *ATG16L1*, were not significantly changed ( Figure 4C). The expression of p62 was opposite to that of ULK1 ( Figure 3C), further indicating that FTO mainly mediates the m ^6^A demethylation of *ULK1* to promote autophagy during starvation-induced HSC activation.

### FTO enhances autophagy and activation in HSCs by targeting ULK1

**Figure 5:**
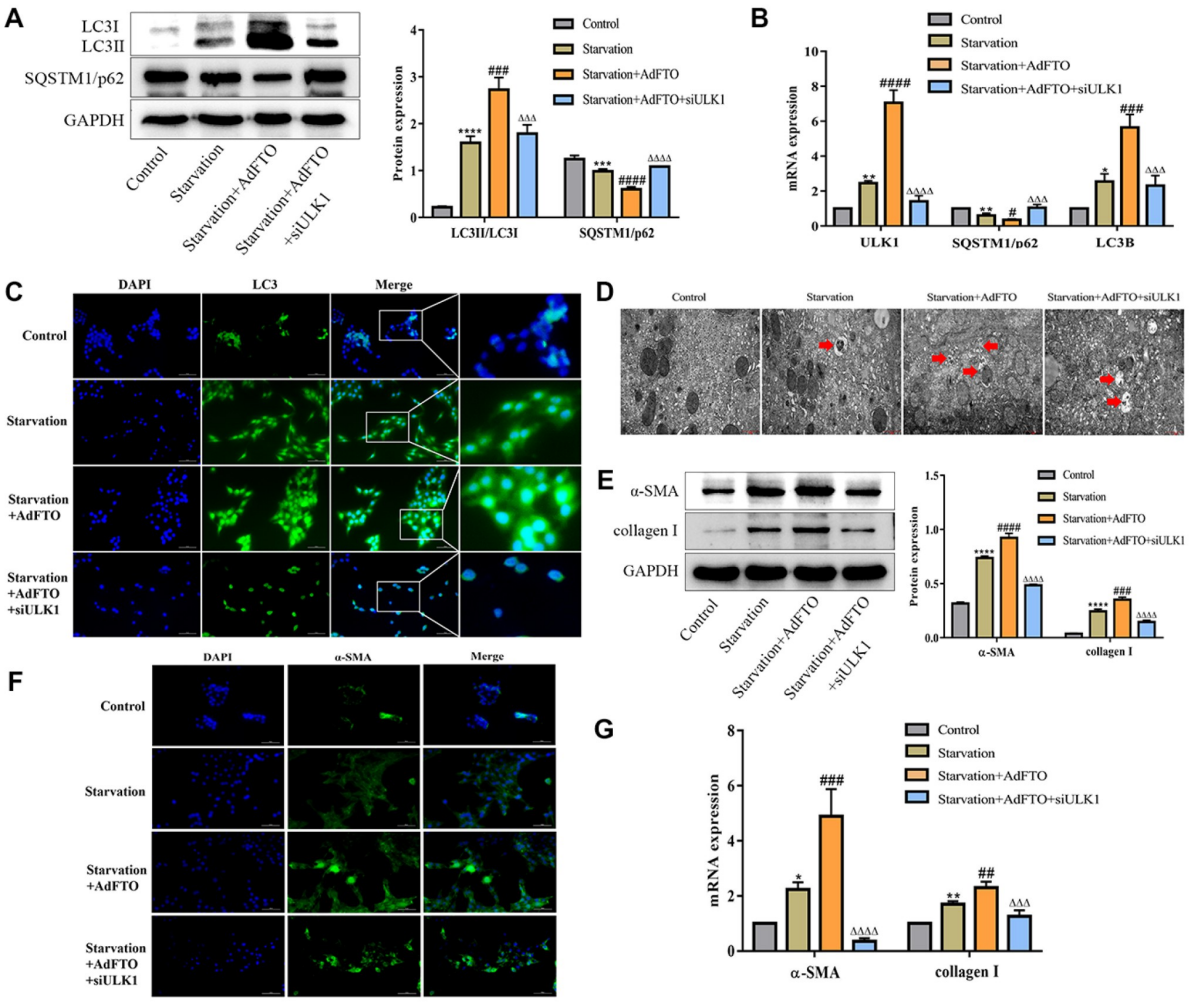
Figure 5 FTO promotes autophagy and HSC activation by targeting ULK1 (A,B) JS-1 cells treated with ULK1 siRNA were transfected with AdFTO for 24 h and then subjected to starvation. The protein and mRNA levels of autophagy markers were measured by western blot analysis and RT-qPCR. ** P<0.01 vs Control, ## P<0.01 vs Starvation, ΔΔ P<0.01 vs Starvation+AdFTO. (C,F) JS-1 cells treated with ULK1 siRNA were transfected with AdFTO for 24 h and then subjected to starvation. Immunofluorescence was used to detect LC3B and α-SMA. The cells were stained for LC3B (green), α-SMA (green) and DAPI (blue). Scale bar: 20 μm. (D) Transmission electron microscopy was used to assess autophagosomes. Scale bar: 1 μm. (E,G) JS-1 cells treated with ULK1 siRNA were transfected with AdFTO for 24 h and then subjected to starvation. The protein and mRNA levels of α-SMA and collagen I were measured by western blot analysis and RT-qPCR, respectively. Data are presented as the mean±SEM, n=3 in vitro. ** P<0.01 vs Control, ## P<0.01 vs Starvation, ΔΔ P<0.01 vs Starvation+AdFTO.

To confirm whether FTO promotes autophagy and HSC activation by affecting the expression of ULK1, we performed a rescue experiment and observed that silencing of *ULK1* reversed the increase in the LC3 II/LC3 I ratio and the decrease in the protein expression of p62 in FTO-overexpressing JS-1 cells followed by starvation ( Figure 5A). Similar to the western blot analysis results, the RT‒qPCR results were the same ( Figure 5B). Consistent with these findings, silencing of *ULK1* significantly reduced the increase in the number of LC3 puncta induced by FTO, as determined by immunofluorescence assays ( Figure 5C). Moreover, compared with that in the starvation+AdFTO group, the number of autophagosomes in the starvation+AdFTO group treated with ULK1 siRNA was decreased ( Figure 5D). These results demonstrated that FTO regulates autophagy by targeting ULK1.

Next, we investigated whether ULK1 is involved in FTO-induced HSC activation. As expected, the increases in the protein expressions of α-SMA and collagen I induced by FTO were decreased upon *ULK1* knockdown ( Figure 5E). In addition, knocking down *ULK1* decreased the increase in the number of α-SMA puncta enhanced by FTO, as determined by immunofluorescence assays ( Figure 5F). Consistently, the mRNA levels of α-SMA and collagen I upregulated by FTO were decreased upon *ULK1* knockdown ( Figure 5G). Taken together, these results illustrated that FTO targeted ULK1, mediated its expression in an m ^6^A-dependent manner, and further regulated HSC autophagy and activation.

### YTHDC2 regulates ULK1 mRNA translation by recognizing the m ^6^A binding site and further affects HSC autophagy and activation

**Figure 6:**
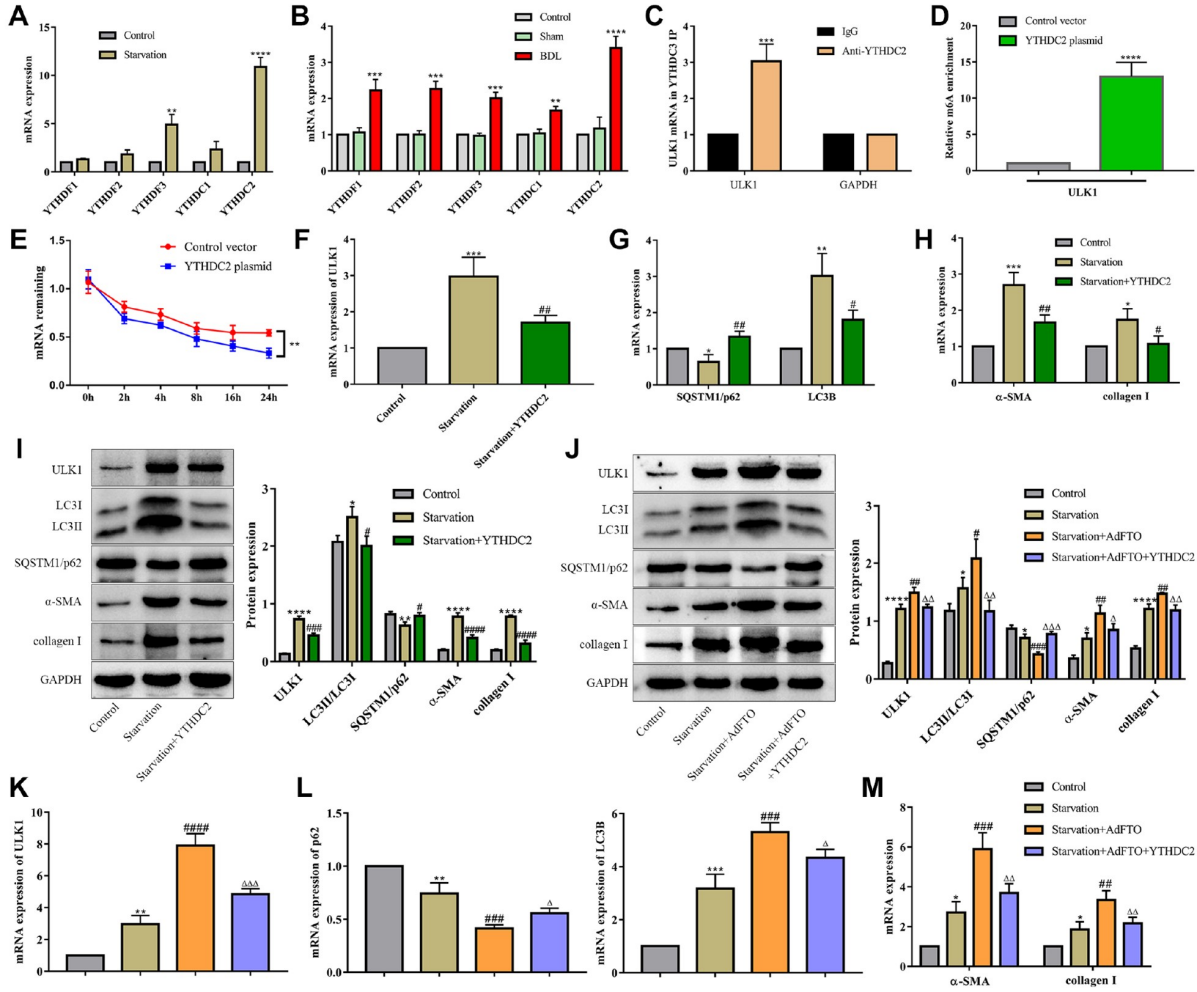
Figure 6 YTHDC2 regulates the expression of ULK1 and HSC activation in a m6A-dependent manner (A, B) mRNA levels of m6A readers were detected by RT-qPCR in vitro and in vivo. ** P<0.01 vs Sham. (C) The binding of YTHDC2 and ULK1 mRNA was measured by RNA immunoprecipitation (RIP). ** P<0.01 vs the IgG group. (D) MeRIP-qPCR analysis of the m6A levels of ULK1 mRNA in the control and YTHDC2 plasmids. ** P<0.01 vs control. (E) RNA stability of ULK1 in the control and YTHDC2 plasmids. ** P<0.01 vs control. (F‒I) mRNA and protein expressions of ULK1, LC3B, SQSTM1/p62, α-SMA and collagen I in starvation-induced JS-1 cells transfected with the YTHDC2 plasmid. ** P<0.01 vs control, ## P<0.01 vs starvation. (J-M) JS-1 cells were transfected with the YTHDC2 plasmid and AdFTO for 24 h and then subjected to starvation. The protein and mRNA levels of ULK1, LC3B, SQSTM1/p62, α-SMA and collagen I were measured by western blot analysis and RT-qPCR. ** P<0.01 vs Control, ## P<0.01 vs Starvation, ΔΔ P<0.01 vs Starvation+AdFTO. Data are presented as the mean±SEM, n=3 in vitro.

YTH domain-containing proteins, including YTHDF1-3 and YTHDC1-2, participate in numerous RNA processes, such as mRNA splicing, nuclear export, translation and decay, via posttranscriptional regulation. To further determine which YTH domain-containing protein mediates the regulation of autophagy by m ^6^A modification, we preliminarily explored the expressions of YTHDF1-3 and YTHDC1-2 in HSC activation and in BDL-induced hepatic fibrosis. The results showed that the expression of YTHDC2 mRNA, but not that of YTHDF1-3 or YTHDC1, increased most significantly both *in vitro* and *in vivo* ( Figure 6A,B). YTH domain-containing proteins play important roles in posttranscriptional modification processes, hence modulating the expression of target genes involved in processes such as autophagy [15]. To verify whether YTHDC2 binds to ULK1 mRNA in HSC JS-1 cells, RNA immunoprecipitation (RIP) was performed. As shown in Figure 6C, compared with those of the *GAPDH* PCR product, the *ULK1* PCR products were highly enriched in YTHDC2, indicating the direct binding of YTHDC2 to *ULK1* mRNA. Furthermore, MeRIP-qPCR revealed that the mRNA transcripts of *ULK1* were significantly enriched by YTHDC2 ( Figure 6D). In addition, an actinomycin D assay showed that YTHDC2 reduced the stability of *ULK1* mRNA ( Figure 6E). Importantly, the expression levels of *ULK1* mRNA and protein were significantly decreased by YTHDC2 ( Figure 6F,I), which is related to the translational function of YTHDC2 in regulating ULK1.

To confirm the effect of YTHDC2 on HSC autophagy and activation, a YTHDC2 plasmid was transfected into JS-1 cells followed by starvation. The results revealed that YTHDC2 downregulated the mRNA and protein expression levels of LC3 and upregulated p62 expression ( Figure 6G,I). As expected, the levels of α-SMA and collagen I were markedly decreased by YTHDC2 ( Figure 6H,I). To further prove that the m ^6^A modification of ULK1 recognized by YTHDC2 is related to FTO, HSCs were transfected with the YTHDC2 plasmid followed by FTO. The results showed that YTHDC2 attenuated the upregulation of ULK1 induced by FTO ( Figure 6J,K). Similarly, the increases in autophagy and HSC activation induced by FTO were reversed by YTHDC2 ( Figure 6J,L, M). Collectively, these data demonstrated that YTHDC2 reduces ULK1 mRNA expression by recognizing the m ^6^A binding site and further decreases HSC autophagy and activation.

## Discussion

A hepatic fibrosis model was induced through BDL. H&E and Masson staining and high expression of α-SMA showed that the liver fibrosis model was successfully established. Accumulating evidence has shown that m ^6^A-related enzymes regulate liver fibrosis; for example, Chai *et al*. [16] revealed that Mettl3 deficiency attenuated HSC activation and CCl _4_-induced liver fibrosis, and Liu *et al*. [17] reported that the m ^6^A reader YTHDF3 alleviated CCl4-induced liver fibrosis. Furthermore, Li *et al*. [18] indicated that exenatide significantly reversed high-fat-induced lipid accumulation and inflammatory changes accompanied by decreased FTO expression. In this study, we revealed that compared with other methylases, FTO, which is the m ^6^A methylase with the most significant difference in expression, was upregulated during HSC activation and BDL-induced hepatic fibrosis. In addition, the region with upregulated FTO was highly coincident with HSC regions, indicating that the increase in FTO is responsible for HSC activation and cholestatic hepatic fibrosis.

The activation of HSCs is central to the occurrence and development of liver fibrosis. m ^6^A modification has been reported to play a critical role in HSC activation [19]. Interestingly, *Shen et al*. [20] recently reported that overexpression of FTO markedly suppressed dihydroartemisinin (DHA)-induced ferroptosis, thus promoting HSC activation. Importantly, our study first revealed that FTO overexpression aggravated HSC activation and hepatic fibrosis, whereas *FTO* knockdown markedly inhibited HSC activation and hepatic fibrosis. Furthermore, previous studies have shown that autophagy is involved in the activation of HSCs in cholestatic liver fibrosis [11]. To clarify the underlying regulatory mechanisms of FTO on HSC activation and liver fibrosis, autophagy was detected. Li *et al*. [20] showed that overexpression of FTO reduced DHA-induced autophagy. However, in this study, we demonstrated that FTO upregulated the levels of autophagy in HSCs and ultimately increased the expressions of activation markers in HSCs through autophagy, which was consistent with our previous finding that aggravated autophagy promoted HSC activation [10]. Therefore, downregulation of FTO-mediated increases in m ^6^A modifications may be a valid method for inhibiting HSC activation.

m ^6^A is the most prevalent posttranscriptional modification of mRNA, occurring at approximately three to five sites per mRNA in mammals [21]. Accumulating studies have indicated that m ^6^A modification participates in autophagy activation by modifying ATGs and further regulates a variety of physiological and pathological processes [22]. Interestingly, Shen *et al*. [23] demonstrated that Mettl14 regulates osteoclast differentiation by inducing autophagy through the m6A/IGF2BP/Beclin1 signaling axis. Moreover, Lee *et al*. [24] revealed that METTL3 inhibits the apoptosis and autophagy of chondrocytes during inflammation through mediating the m6A/YTHDF1/Bcl2 signaling axis. In the present study, we found that ULK1 is rich in methylation sites due to increased m ^6^A modification caused by knockdown of the demethylase gene *FTO*. Furthermore, overexpression of FTO significantly upregulated the level of ULK1, while there were no significant differences in the other ATGs. Our study suggested for the first time that FTO mainly mediates the m ^6^A demethylation of ULK1 to drive autophagy.

As a protein kinase, ULK1 is activated upon autophagy stimulation and is critical for recruiting other autophagy-related proteins to autophagosome formation sites [25]. He *et al*. [26] verified that the ULK1 activator BL-918 has therapeutic potential for amyotrophic lateral sclerosis through the induction of cytoprotective autophagy. Based on accumulating evidence, we further confirmed the role of ULK1 in FTO-induced autophagy and activation of HSCs. Indeed, our data suggested that ULK1 is positively correlated with HSC autophagy and activation. More importantly, *ULK1* knockdown reduced the formation of autophagosomes and ECM accumulation, which were markedly increased by FTO. These results provide evidence that FTO enhances autophagy and the activation of HSCs by targeting ULK1.

Recently, m ^6^A reader proteins were found to participate in posttranscriptional modification processes, thus regulating the expressions of genes related to inflammation and autophagy [15]. According to one report, the m ^6^A reader protein YTHDF1 regulates autophagy by targeting SQSTM1 in diabetic skin [27]. In our study, YTHDC2 is the most significantly differentially expressed gene among genes in the YTH family involved in HSC activation and cholestatic liver fibrosis. We confirmed that overexpression of YTHDC2 decreased ULK1 level by recognizing the m ^6^A binding site and ultimately inhibited autophagy and HSC activation. More importantly, YTHDC2 weakened the increase in ULK1 induced by FTO, thereby improving autophagy and HSC activation induced by FTO. However, the mechanism by which YTHDC2 modulates ULK1, thus affecting HSC activation, needs to be further explored.

In summary, this study demonstrated that the m ^6^A demethylase FTO promotes HSC autophagy by targeting ULK1, resulting in HSC activation and ultimately leading to hepatic fibrosis. Furthermore, the m ^6^A reader YTHDC2 was shown to specifically regulate ULK1. Our findings provide new insight showing that targeting the FTO/ULK1 axis may be a promising strategy for clinical anti-liver fibrosis therapy.
